# Synthesis and photoluminescence properties of ZnS nanobowl arrays via colloidal monolayer template

**DOI:** 10.1186/1556-276X-9-389

**Published:** 2014-08-11

**Authors:** Yanping Liu, Zhigang Li, Wenwu Zhong, Li Zhang, Weiping Chen, Qintao Li

**Affiliations:** 1 Department of Physics and Electronic Engineering, Taizhou University, Taizhou, Zhejiang 318000, China

**Keywords:** Colloidal monolayer template, ZnS nanobowl arrays, Photoluminescence

## Abstract

Two-dimensional Zinc sulfide (ZnS) nanobowl arrays were synthesized via self-assembled monolayer polystyrene sphere template floating on precursor solution surface. A facile approach was proposed to investigate the morphology evolution of nanobowl arrays by post-annealing procedure. Photoluminescence (PL) measurement of as-grown nanoarrays shows that the spectrum mainly includes two parts: a purple emission peak at 382 nm and a broad blue emission band centering at 410 nm with a shoulder around 459 nm, and a blue emission band at 440 nm was obtained after the annealing procedure. ZnS nanoarrays with special morphologies and PL emission are benefits to their promising application in novel photoluminescence nanodevice.

## Background

Two-dimensional (2D) micro- and nanostructured arrays have attracted much interest because of their potential applications in optoelectronic device, gas sensors, solar cell, self-cleaning surface, etc. [1-5]. The applications and physical properties of 2D nanoarrays are relevant to their morphology. For example, bowl-like nanostructural arrays can be used in various fields, like nanoscale container and size selection of submicron spheres [6], light emitting diode surface [7], and chemical and biological sensors [8]. Therefore, the control of nanoarrays' morphology provides opportunities to tune and improve their properties and further promote their practical applications in various fields.

Zinc sulfide (ZnS), as one of direct band gap semiconductor material, is a luminescent material well-known for its photoluminescence (PL) and electroluminescence [9,10], which enables wide applications in the fields of flat-panel display [11], sensor and lasers [12,13], and photodetectors [14]. Up to date, many efforts have been focused on the morphologies control and PL emissions of ZnS nanomaterials [15-20]. It was demonstrated that their PL emissions were dominated by defect states, such as surface states, stoichiometric vacancies, and interstitial lattice defects [16-19]. However, the defect states in ZnS nanomaterial are active, especially at surface site, which will lead to unstable physical properties as well as poor reliability of ZnS-based nanodevices. It had been reported that the PL intensity of ZnS quantum dots diminished after a few days when left in the condition of normal laboratory lighting [21]. Therefore, such active defects in ZnS nanomaterials should be post-treated to keep the stable properties before practical applications. Post-annealing may be a facile and effective method to solve this problem. Defect states at material surface will be greatly reduced with the increased grain size after annealing, while the interstitial lattice defects tend to stabilize. On the other hand, the morphology of ZnS nanomaterials could be changed with the increased grain size, which may provide a facile approach to control the morphology of nanomaterials by post-treatment. It would be desirable to obtain the ZnS nanomaterials with controlled morphology and stable properties by annealing, which is favorable for their special applications.

In recent years, monolayer colloidal crystal template (MCCT) has proved to be an effective approach for fabricating ordered nanoarrays due to their low cost, flexibility, and various morphologies [22,23]. Moreover, MCCT exhibits excellent compatibility with traditional fabrication technologies, such as sol-gel [24,25], pulse laser deposition [26], vapor deposition [27], and electrochemical deposition [28-30]. It is worth noting that the nanosphere lithography at the solution surface (NSLSS) had been developed by Qi's group [8,31,32], which provides a practical route for fabricating high-quality 2D nanoarrays with special morphologies.

In this work, we devoted to the morphology evolution and the PL emission of 2D ZnS nanobowl arrays through selecting the annealing ambient. The 2D ZnS nanobowl arrays were fabricated by NSLSS method at water bath. The special morphologies of nanoarrays can be obtained after annealing in different ambients, while the surface defect-related PL emissions can be prohibited or suppressed, leaving a blue emission band at around 440 nm. The results indicate that the 2D ZnS nanobowl arrays with special morphology may promote their application in PL-related nanodevice and further broaden their promising application in the field of flexible nanoscale device and bionic optical devices.

## Methods

Prior to preparation of MCCT, glass substrates were ultrasonically cleaned with acetone, ethanol, 98% H_2_SO_4_:H_2_O_2_ (3:1), H_2_O:NH_3_ · H_2_O:H_2_O_2_ (5:1:1), and distilled water for 60 min, respectively. Polystyrene spheres of 600 nm (10 wt%) employed in the experiment was purchased from Duke Scientific Corporation (Pudong, Shanghai, China). MCCT was formed by a gas-liquid interface self-assembly method, which was discussed in detail in our previous work [33]. In brief, a cleaned glass substrate was fixed at the center of a Petri dish surrounded by distilled water. Then, a drop of 10 μl water-ethanol-diluted PS suspension solution was dropped on the glass surface, and the PS suspension spreads freely and self-assembles into a colorful MCCT film on the water surface. The MCCT was lifted by a silicon substrate and the residual water around the MCCT was absorbed by a filter paper, which was used as a template for synthesizing nanoarrays on the solution surface.

**Figure 1:**
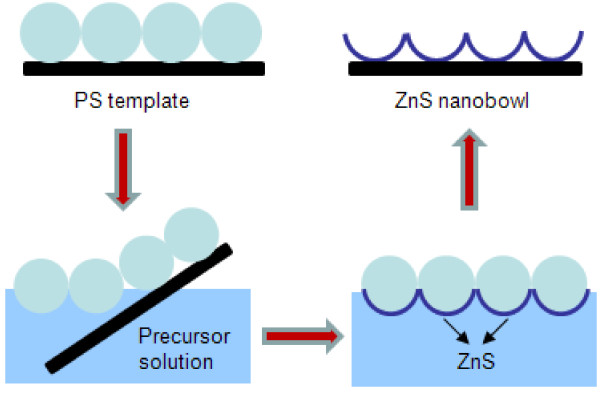
Illustration of the synthesis route of ZnS nanobowl arrays.

Usually, ZnS nanomaterials were synthesized in a water solution system at low temperature [8,18]. In a typical reaction, 1.2 ml of 1 M zinc acetate solution [Zn(CH_3_COO)_2_ · 2H_2_O, ZnAc], 4 ml of 1 M ammonium acetate (CH_3_COONH_4_ · 2H_2_O), and 3 ml of 0.2 M disodium ethylenediamine tetraacetic acid (Na_2_EDTA) were mixed in a vessel, and then 4.8 ml of 0.5 M thioacetamide (TAA) was added. Subsequently, 7 ml of distilled water was added into the mixed solution to reach a total volume of 20 ml. Then, the MCCT-covered silicon substrate was inserted in the mixed solution with a tilted angle, and the MCCT was peeled from silicon substrate and floated on the surface of the mixed solution, as shown in Figure 1. The vessel was placed in the water bath at 60°C for 6 h. Then, the MCCT floated on the mixed solution surface was lifted by silicon or glass substrate, and the PS spheres were dissolved in dichloromethane to obtain ZnS nanoarrays on the substrate. The obtained ZnS nanoarrays were annealed in muffle furnace, magnetron sputtering device with a vacuum of 10^−4^ Pa, and quartz tube with a constant argon flow of 400 ml/min (argon/hydrogen = 95:5) at 300°C for 2 h, respectively.

The nanoarrays were characterized using field emission scanning electronic microscope (FESEM) and energy dispersive X-ray spectroscopy (EDS) (Hitachi S-4800, Hitachi, Tokyo, Japan), X-ray diffractometer (XRD, Bruker AXS D8 Advance, Bruker Corporation, Karlsruhe, Baden-Württemberg, Germany), and photoluminescence spectrometer (Hitachi F-4600) using 325 nm as the excitation wavelength, respectively.

## Results and discussion

**Figure 2:**
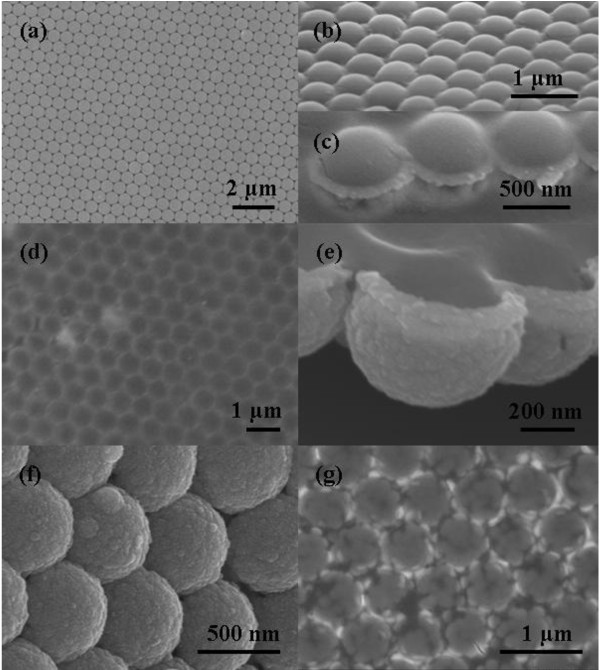
**SEM images of MCCT and ZnS nanobowl arrays. (a)** MCCT made of 600 nm PS spheres. **(b-f)** ZnS nanobowl arrays after 6 h reaction time: **(b, c)** side view of ZnS nanobowl arrays with MCCT, **(d)** top view, and **(e, f)** side view of ZnS nanobowl arrays. **(g)** Top view of ZnS nanobowl arrays after 3 h reaction time.

**Figure 3:**
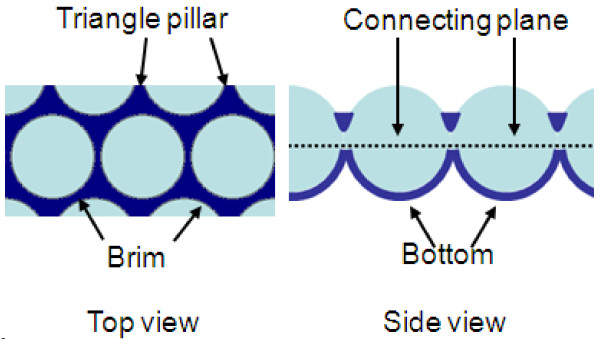
Illustration of the structure for ZnS nanobowl arrays.

A large-area PS MCCT of 600 nm with hexagonal close-packed arrangement was employed to fabricate the ordered ZnS nanobowl arrays (Figure 2a). It can be clearly seen that ZnS nanoparticles filled the triangle interstices among the PS spheres (Figure 2b), and the ZnS particles were deposited both at the bottom and upside of PS spheres after reacting 6 h at solution surface, as shown in Figure 2c. The top view shows highly ordered inverse opal pore-like arrays after removing the PS template (Figure 2d). The center of two neighboring pores is equal to the diameter of PS spheres (600 nm), indicating that the inverse nanostructural arrays remain the periodicity of PS template. As shown in Figure 2e, f, the side view of high-magnification SEM images exhibits that the units of arrays possess completed side wall and closed bottom, revealing that the bowl-like arrays were formed in experiments. The as-grown nanobowls consist of approximately 10 nm ZnS nanoparticles, and the height and the wall thickness of nanobowl are about 340 and 65 nm, respectively. It is noted that the height of the nanobowl is slightly larger than the radius of PS spheres (300 nm), indicating that ZnS nanoparticles were deposited at the upper hemisphere and connected to a circular unit from the top view of SEM (Figure 2d). For a shorter time of 3 h, most nanobowls exhibit unclosed wall as well as the part discrete triangle pillars distributed at the top of bowls, resulting in a discontinuous brim of nanobowl arrays, as shown in Figure 2g. These results indicate that the wall thickness and connection of nanobowl are time-dependent. In a typical NSLSS method, the nanoparticles nucleate and grow at PS-solution interfaces [8]. Therefore, ZnS nanoparticles tend to connect each other to form a continuous film around PS spheres with the prolonged reaction time (Figure 2c). The reaction time should be prolonged to 6 h to form a complete bowl arrays in experiments.To clarify the morphology evaluation of ZnS nanobowl, it was divided into three parts: bottom, triangle pillar, and upper brim, as shown in Figure 3. As mentioned above, the height of nanobowl is larger than the radius of PS sphere, and the nanoarrays deposited above connecting plane are fragile, especially at the brim of bowls, after removing the PS template. Therefore, the morphologies of the arrays could be changed by certain post-treatment process.

**Figure 4:**
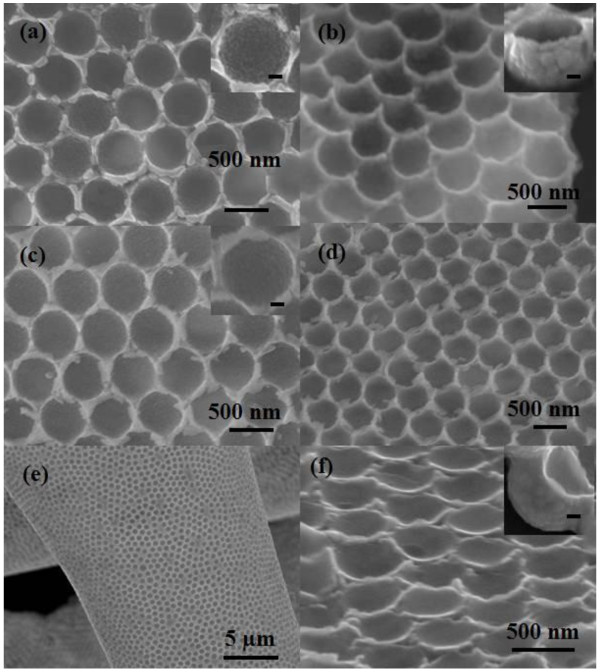
**SEM images of ZnS nanobowl arrays annealed in different ambients. (a, b)** Air, **(c-e)** vacuum, and **(f)** argon. **(a, c, e)** Top view and **(b, d, f)** side view. The insets of **(a-c, f)** show the magnification images of individual ZnS nanobowl. The scale bar is 100 nm for all the insets.

Figure 4 shows the SEM images of nanobowl arrays annealed in different ambients at 300°C for 2 h. As shown in Figure 4a,b, the upper brim of the bowl is absent and the triangle pillars remain in the nanoarrays after annealed in air, leaving the convex triangle pillars connected by sunken brim. It is also shown that both the inner and outer wall of nanobowls become rough, and the size of ZnS nanoparticles increases to approximately 35 nm, revealing that the crystallization of nanoparticles was improved by annealing. When annealed in vacuum, as shown in Figure 4c,d, both the triangular pillars and the upper brims are present in nanoarrays, and even part of upper brims was broken during annealing. Moreover, the nanobowl also exhibits a rough inner wall made of approximately 20 nm ZnS nanoparticles. In addition, the ZnS nanoarray film also shows good flexibility when the film was peeled from the silicon substrate (Figure 4e), which may provide a promising application in flexible nanodevice. In the case of annealing in argon, a concave nanostructural array is obtained, and either the convex triangle pillars or the upper brim of nanobowl is not observed in the arrays, while the bowl shows a relatively smooth inner wall, indicating the poor crystallization of ZnS nanoparticles. It has been reported that the blue structural colors of *Papilio* butterfly wings originate from the mixed reflections of blue and yellow colors on a multilayer stacking concavity microstructure [34]. Therefore, such concave structure would be a good candidate for the promising application in bionic optical devices.

**Figure 5:**
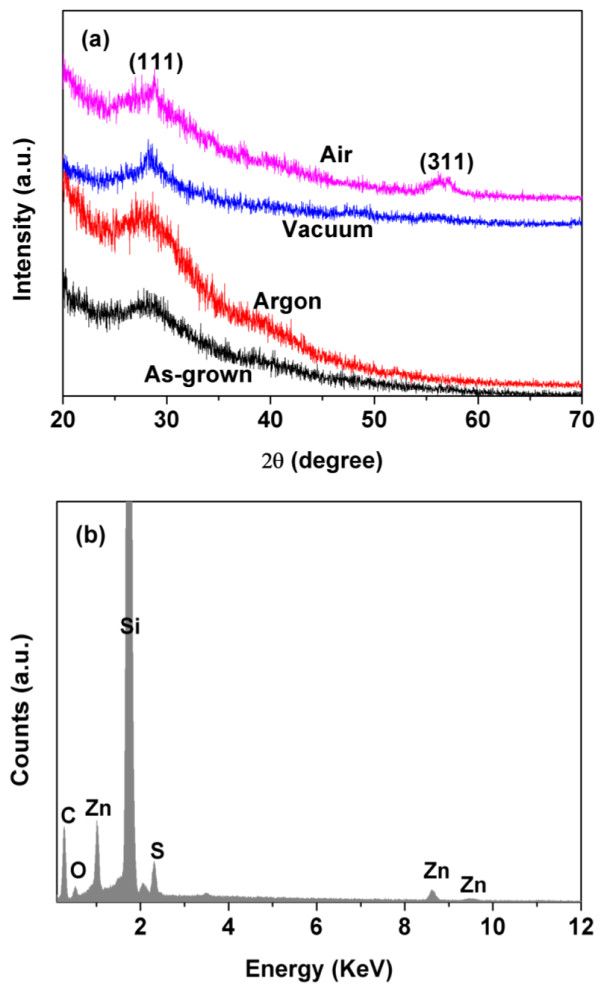
**XRD patterns and EDS spectrum of ZnS nanobowl arrays. (a)** XRD patterns of ZnS nanobowl arrays annealed in different ambients. **(b)** EDS spectrum of as-grown ZnS nanobowl arrays.

These results reveal that the upper brims of the bowl play an important role in determining the morphologies of nanobowl arrays, which are gradually broken with the increasing size of ZnS nanoparticles, resulting in different morphologies in air and vacuum. However, in the case of argon, the absence of nanoarrays above the connecting plane, including the triangle pillars and upper brim of the bowl, is independent on their poor crystallization, which was destroyed by the constant argon flow.Figure 5a shows the XRD patterns of ZnS nanobowl arrays annealed in different ambients. The diffraction peaks of 28.5° and 56.3° were indexed as (111) and (311) planes of the cubic zinc blend structure (JCPDS No. 05-0566). XRD results show a polycrystalline structure for ZnS nanoarrays. For all samples, the observed diffraction peaks were quite broad, indicating the nanoarrays made of nanosize particles. Unfortunately, the average size of the nanoparticles could not be estimated by Scherrer equation due to the weak diffraction signals. There is not much difference among the intensity of the (111) peak for all the samples. In general, the intensity of (111) peak is slightly increased after annealing. However, the (111) peak becomes narrow as annealed in vacuum and a new peak (311) presents as annealed in air, indicating the improvement of crystallization as well as the increase of nanoparticles size, while the nanoarray reveals the poor crystallization in argon, which agrees with the SEM results.The chemical composition of the as-grown ZnS nanoarrays was characterized by EDS, as shown in Figure 5b. The atomic ratio of Zn:S is about 1:0.9, which slightly deviates from their stoichiometric ratio. The signals of Si, O, and C in the EDS spectrum were ascribed to silicon substrate and contamination of decomposed PS spheres, respectively.

**Figure 6:**
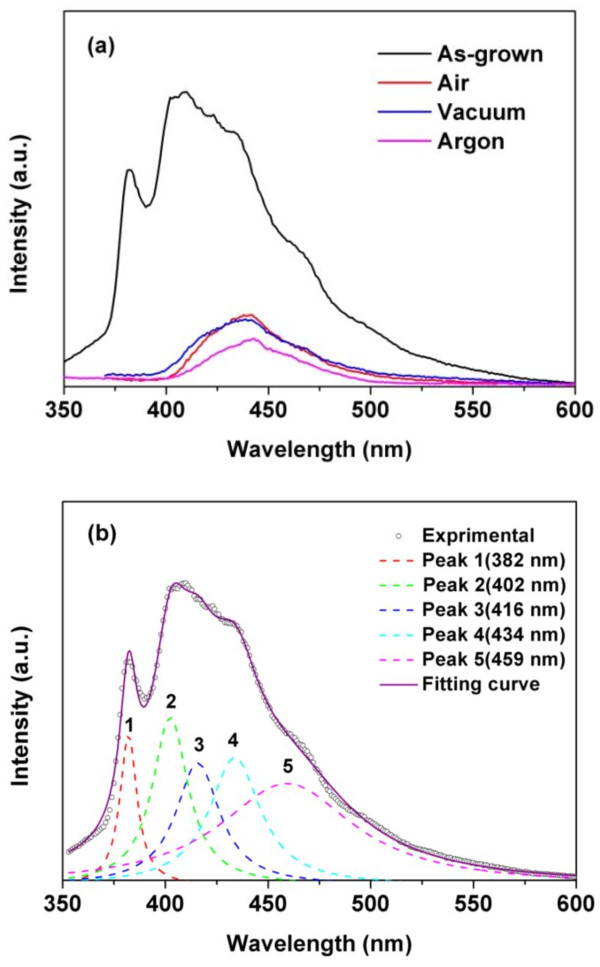
**PL emission spectra and multipeak Lorentzian fit of the as-grown nanoarrays.** PL emission spectrums of ZnS nanobowl arrays annealed in different ambients **(a)** and multipeak Lorentzian fit of the as-grown nanoarrays **(b)**.

The PL measurement of the ZnS nanoarrays was carried out at room temperature with 325 nm excitation. As shown in Figure 6a, a multipeak emission is observed in as-grown nanoarrays, including a purple emission band at 382 nm and a broad blue emission band centered at 410 nm with a weak shoulder around 460 nm. Unlike those reported characteristic emission of metal-doped luminescent nanomaterials [35,36], these compound PL spectra with broad feature are assigned to different species defect states in ZnS nanocrystallites, which will be discussed in following section. The PL spectra of nanoarrays annealed in different ambients show an almost identical nature, located in the blue region with its maximum intensity centered at 440 nm. A multipeak Lorentzian fit of the as-grown nanoarrays gives five emission bands located at 382, 402, 416, 434, and 459 nm, respectively. As shown in Figure 6b, the Lorentzian curves fit well with the experimental data.

As well known, the PL emissions of ZnS nanomaterial are closely dependent on their morphologies and the preparation parameters due to their large surface-to-volume ratio. In general, the purple and blue emissions are ascribed to the defect states in ZnS nanomaterials, such as stoichiometric vacancies, interstitial lattice defects, and surface states. Among these defects, interstitial zinc and sulfur vacancies act as shallow donors (electron traps) and interstitial sulfur and zinc vacancies can behave as deep acceptors (hole traps) [37]. The ascending order of the defect-related emission wavelength is interstitial sulfur, interstitial zinc, sulfur vacancies, and zinc vacancies [38]. In our experiment, zinc vacancies-related luminescence is not observed, which is often located about 480 nm [39]. Therefore, the emission peaks at 382 and 402 nm could be assigned to the radiative recombination of interstitial sulfur, and the peaks at 416 and 434 nm are attributed to interstitial zinc and sulfur vacancies, respectively. The interstitial defects are always present at the surface of ZnS nanomaterials prepared by chemical methods. In addition, the peak at 459 nm is associated with the trapped luminescence arising from the surface state [16].

For explaining the quenched emission peaks (at 382, 402, 416, and 459 nm) and the decreased emission intensity of 440 nm, the effects of annealing on the PL emission are proposed. Firstly, the interstitial defects at the nanobowl surface site, including interstitial zinc and interstitial sulfur, have diffused out the lattice due to the sufficient drive force originating from the annealing process. Hence, the corresponding PL emissions (at 382, 402, and 416 nm) are quenched. Zeng et al. [40] reported the similar results in ZnO nanomaterials; they believed that the annealing induces the outward diffusion of interstitial zinc and quenching of blue emissions. Secondly, the increased size and the improved crystallization of ZnS nanoparticles after annealing in air and vacuum, as evidenced by SEM and XRD results, will lead to the decrease of their surface area as well as the disappearance of a large number of surface states. Therefore, the surface states-related PL emissions (at 459 nm) were prohibited or attenuated. In the case of argon, the surface states should be passivated by the presence of hydrogen as mentioned in experimental section, and hence their surface defect-related PL emissions (at 459 nm) were vanished. It had been approved that defects at the surface site of ZnS nanomaterials were easily occupied by other impurity atoms, and the corresponding defect-related emission was quenched [41]. The passivation of defect states was usually employed to investigate the luminescence character of nanomaterials [42,43]. According to the above results, the lower emission intensity (at 440 nm) of the annealed samples could be easily understood. The PL spectra of as-grown sample are compound emissions of different species defects, and the emissions of interstitial defects and surface states are quenched by annealing, which are mainly responsible for the decreased emission intensity. On the other hand, the improved crystal structure (in air and vacuum) or defect-passivated (in argon) ZnS nanomaterials induced by annealing should also lead to the decrease of the defect-related emission intensity. Comparing with the sulfur vacancy-related emission (at 434 nm) of the as-grown sample, a red shift of 6 nm (from 434 to 440 nm) could be arising from the fit error or the changed surrounding of defects after annealing.

These results indicate that a large number of defect states located at the surface site of as-grown ZnS nanobowl and such defect-related PL emissions are unstable, which could be eliminated by annealing. Moreover, a blue emission band around 440 nm obtained from different annealing ambients is very stable, and its intensity is unchanged when the samples are kept in air for several months.

## Conclusions

The 2D nanobowl arrays of ZnS were synthesized via a MCCT floating on the precursor solution surface. A facile post-annealing was proposed to investigate the morphology evolution and PL emissions of ZnS nanobowl arrays. The results indicate that various morphologies of ZnS nanobowl arrays could be obtained by annealing in different ambients. The surface defect-related PL emissions were suppressed, while a stable blue emission was obtained after annealing. ZnS nanobowl arrays with special morphologies and stable PL emission are essential to ensure the reliability of PL-based nanodevice and further accelerate their practical applications in various fields. In addition, the morphologies obtained in different annealing ambients may be a promising candidate in the field of flexible nanodevice and bionic optical devices.

## Competing interests

The authors declare that they have no competing interests.

## Authors’ contributions

YL carried out the synthesis process, analyzed the data, and drafted the manuscript. ZL and WC proposed the initial work, supervised the experimental work, and revised the manuscript. WZ and LZ participated in SEM imaging and image analysis. QL participated in PL emission analysis. All authors read and approved the final manuscript.
